# Effects of Anesthetic Technique on Outcomes After Hip Fracture Surgery in Geriatric Patients: A Retrospective Observational Study

**DOI:** 10.7759/cureus.111436

**Published:** 2026-06-24

**Authors:** Yudhyavir Singh, Aayush Kulshrestha, Abhishek Singh, Riniki Sarma, Sharmishtha Pathak, Chhavi Sawhney, Babita Gupta

**Affiliations:** 1 Anesthesiology, All India Institute of Medical Sciences, New Delhi, New Delhi, IND

**Keywords:** anesthetic technique, combined spinal epidural anesthesia, general anesthesia, geriatric hip fracture, subarachnoid block

## Abstract

Background: Hip fractures in older adults almost always require surgery under general or neuraxial anesthesia. While the influence of anesthetic technique on mortality has been studied extensively, its relationship with resource-related outcomes such as hospital length of stay is less well characterized, particularly in high-volume trauma settings.

Objective: To determine whether anesthetic technique is associated with hospital length of stay in geriatric patients undergoing hip fracture surgery (primary objective). Secondary objectives were to compare ICU admission and length of stay, and postoperative adverse events across techniques, and to identify patient comorbidities associated with a prolonged hospital stay.

Materials and methods: This single-center retrospective observational study included 265 consecutive patients aged ≥65 years undergoing acute hip fracture surgery between April 2022 and March 2023. Patients were grouped by anesthetic technique: subarachnoid block (SAB), combined spinal-epidural anesthesia (CSE), or general anesthesia (GA). Length of stay was compared across techniques using the Kruskal-Wallis test with Dunn’s post hoc comparisons; ICU admission and adverse events using Fisher’s exact test; and comorbidity-length-of-stay associations using the Mann-Whitney U test. A multivariable negative-binomial model estimated adjusted incidence-rate ratios (IRRs).

Results: The median hospital length of stay was five days (interquartile range 3-7) and differed significantly by technique (SAB four days, GA six days, CSE nine days; p < 0.001), a gradient that persisted after adjustment (adjusted IRR versus SAB: CSE 2.84 (95% CI 2.51-3.22), GA 1.72 (1.44-2.05)). ICU admission (CSE 13.2%, GA 6.1%, SAB 2.6%; p = 0.006) and any postoperative adverse event (CSE 17.1%, GA 9.1%, SAB 3.8%; p = 0.002) were likewise highest after CSE. In-hospital mortality (1.5%) and 30-day readmission (1.9%) did not differ by technique. A longer stay was associated with coronary artery disease, renal disease, and a prior stroke.

Conclusions: Hospital length of stay, ICU admission, and postoperative adverse events were greatest among patients receiving CSE anesthesia and least among those receiving SAB, whereas in-hospital mortality did not differ by technique. Given the observational design and the broadly similar baseline characteristics across groups, these differences most likely reflect the indications for each technique and the care pathways associated with it rather than an effect of anesthesia itself. Anesthetic techniques for geriatric hip fracture surgery can therefore be individualized to patient and operative factors, and prospective studies are needed to confirm these associations and clarify their mechanisms.

## Introduction

Hip fractures represent a major and growing global public-health problem; an estimated 9.6 million new cases occurred worldwide in 2019 among adults aged 55 years and older, and the number is projected to rise further as populations age and the prevalence of osteoporosis and frailty increases [1]. The overwhelming majority of hip fractures in older adults require surgical fixation, which in turn necessitates either general or regional anesthesia (RA). Despite advances in perioperative care, outcomes after hip fracture surgery remain poor: one-year mortality is typically 15-30%, fewer than half of survivors regain their pre-fracture functional status, and many experience prolonged hospitalization, functional decline, and loss of independence [2].

Although the determinants of these adverse outcomes are multifactorial, the choice of anesthetic technique is one of the few readily modifiable factors. RA techniques avoid the need for definitive airway management and have a more limited effect on cerebral function but are associated with hypotension and may be unsuitable for patients receiving anticoagulants. General anesthesia (GA) avoids these particular limitations but may adversely affect postoperative mentation. The comparative evidence, however, remains inconsistent [3]. Meta-analyses have variously reported reductions in blood loss, operating time, and deep vein thrombosis with RA, a lower 30-day mortality, a reduction in in-hospital mortality without any difference at 30 days, and no difference in mortality at all, making it difficult to formulate definitive guidelines for anesthetic practice in this population [4-6].

Beyond mortality, the length of hospital and ICU stay has become an increasingly important outcome after hip fracture surgery [7]. It reflects the cumulative burden of perioperative morbidity and the pace of recovery, is a principal determinant of healthcare cost and bed utilization, and is of particular relevance in high-volume, resource-constrained settings. The extent to which the choice of anesthetic technique influences length of stay as distinct from mortality has been less characterized, and the contribution of patient comorbidity to a prolonged stay in this population remains incompletely understood.

We therefore conducted this retrospective observational study in geriatric patients undergoing acute hip fracture surgery. Our primary objective was to determine whether the anesthetic technique is associated with hospital length of stay. The secondary objectives were to compare ICU admission, ICU length of stay, and postoperative adverse events across different anesthetic techniques and to identify patient comorbidities associated with a prolonged hospital stay, thereby distinguishing patient-related contributors to length of stay from the anesthetic technique itself.

## Materials and methods

Study design and setting

This was a single-center, retrospective, observational study conducted at the Jai Prakash Narayan Apex Trauma Centre (JPNATC), All India Institute of Medical Sciences (AIIMS), New Delhi. The study was approved by the Institutional Ethics Committee (approval number: IEC-514/01.9.2023), and in view of its retrospective design, the requirement for individual written informed consent was waived.

Study population and eligibility criteria

Consecutive patients admitted to JPNATC for acute hip fracture surgery between 1 April 2022 and 31 March 2023 were screened for eligibility. Patients were included if they were aged 65 years or older, underwent surgery for a hip fracture (transcervical/neck of femur, intertrochanteric, or subtrochanteric), and received either GA or RA. Eligibility was not restricted by American Society of Anesthesiologists (ASA) physical status; patients of all ASA classes encountered during the study period were included, and the final cohort comprised ASA classes I to IV. Patients were excluded if they had a pathological or bilateral fracture, had undergone previous surgery on the affected hip, had multiple fractures, received combined regional and GA, or had a known allergy to local anesthetics.

Anesthetic technique

The anesthetic technique was chosen by the attending anesthesiologist according to patient factors, surgical requirements, and institutional practice. Single-shot subarachnoid block (SAB) was generally used for shorter, predictable procedures, whereas combined spinal-epidural anesthesia (CSE) was preferred for longer or less predictable surgeries and when postoperative epidural analgesia was planned. GA was used when neuraxial anesthesia was contraindicated, not feasible, or had failed. For analysis, patients were grouped according to the technique received: SAB, CSE, or GA; SAB and CSE together constituted the RA techniques.

Anesthetic administration and perioperative management

All anesthetics were administered or supervised by consultant anesthesiologists in accordance with institutional practice. Standard monitoring included electrocardiography, non-invasive blood pressure, and pulse oximetry for all patients, with capnography and temperature monitoring added during GA.

Neuraxial blocks were performed under aseptic conditions in the sitting position at the L3-L4 or L4-L5 interspace. SAB was achieved using 10 mg of 0.5% hyperbaric bupivacaine with 20 μg fentanyl. For CSE, the spinal component was administered similarly, followed by epidural catheter placement using a needle-through-needle technique to facilitate intraoperative anesthesia and postoperative analgesia. GA was induced with intravenous fentanyl (1-2 μg/kg), propofol (1-2 mg/kg), and rocuronium (0.6 mg/kg). Following endotracheal intubation, anesthesia was maintained with isoflurane, and neuromuscular blockade was reversed with neostigmine.

Perioperative management followed institutional protocols, including preoperative optimization, multimodal analgesia, and early postoperative mobilization. Patients recovered in the post-anesthesia care unit and were subsequently managed on the ward; ICU admission was reserved for those with hemodynamic instability or significant comorbidities requiring close monitoring. Discharge was planned once pain was adequately controlled, assisted mobilization was achieved, and medical stability with appropriate follow-up care was ensured.

Data collection and variables

Data were extracted retrospectively from the hospital medical records, including anesthesia charts, operative notes, nursing records, and discharge summaries, using a standardized data-collection form. Extraction was performed by two investigators, and any discrepancies were resolved by discussion or by referral to a senior author. Outcomes and adverse events were recorded according to a prespecified pro forma, and the extracted data were de-identified before analysis. The variables collected comprised demographic characteristics (age and sex); fracture type; surgical procedure (closed reduction and internal fixation, open reduction and internal fixation, bipolar hemiarthroplasty, or total hip replacement); ASA physical status; injury severity, characterized by the Abbreviated Injury Scale (AIS) [8] and the Injury Severity Score (ISS) [9]; the anesthetic technique; and the burden of comorbidity, recorded as the presence or absence of hypertension, diabetes mellitus, coronary artery disease, chronic obstructive pulmonary disease, prior stroke, renal disease, thyroid disorder, malignancy, dementia, heart failure, atrial fibrillation, asthma, seizure disorder, and liver disease.

The outcomes recorded were the hospital length of stay (days); ICU admission and, where applicable, ICU length of stay (days); in-hospital death; 30-day readmission; and postoperative adverse events, comprising respiratory events, acute kidney injury, surgical site infection, urinary retention, myocardial infarction, delirium, sepsis, and deep vein thrombosis.

Outcome measures

The primary outcome measure was hospital length of stay. The secondary outcome measures were ICU length of stay, postoperative adverse events, and the effect of comorbidity on hospital length of stay.

Statistical analysis

Analyses were performed using Stata version 19.0 (StataCorp LLC, College Station, Texas, USA). The distribution of continuous variables was assessed using the Shapiro-Wilk test together with visual inspection. Normally distributed continuous variables are presented as mean ± standard deviation, and non-normally distributed variables as median with interquartile range (IQR); categorical variables are presented as frequencies and percentages. As the length-of-stay data were markedly skewed, they were summarized as medians (IQR) and analyzed using non-parametric methods. Because of the retrospective nature of the study, all eligible patients during the study period were included, and no formal a priori sample size calculation was performed.

For the primary analysis, hospital length of stay was compared across the three anesthetic techniques (SAB, CSE, and GA) using the Kruskal-Wallis test, with Dunn’s post-hoc test and Bonferroni correction for pairwise comparisons. ICU length of stay was likewise compared across techniques using the Kruskal-Wallis test, where group sizes permitted, and the frequency of ICU admission and of postoperative adverse events were compared across techniques using Fisher’s exact test (Freeman-Halton extension for the three-group comparisons), which was preferred to the chi-square test because of the low frequency of events. The association of each binary comorbidity and of sex with hospital length of stay was assessed using the Mann-Whitney U (Wilcoxon rank-sum) test, and length of stay was compared across ASA physical-status categories using the Kruskal-Wallis test.

To determine whether the association between anesthetic technique and hospital length of stay was independent of patient characteristics, a multivariable negative-binomial regression model was fitted with length of stay (in days) as the outcome and anesthetic technique, age, sex, ASA physical status, fracture type, and the number of comorbidities as covariates. The negative-binomial distribution was preferred to the Poisson because of overdispersion of the length-of-stay data, and the dispersion parameter was estimated by maximum likelihood. Results are expressed as adjusted incidence-rate ratios (IRRs) with 95% confidence intervals, with SAB as the reference category, and a median (quantile) regression model with the same covariates was fitted as a sensitivity analysis. A two-sided p-value of less than 0.05 was considered statistically significant throughout.

## Results

Cohort characteristics

During the one-year study period (April 2022-March 2023), 265 patients aged ≥65 years who underwent acute hip fracture surgery were included. The mean age was 76.7 ± 7.9 years (median 75, IQR 70-82; range 65-98), and there was a slight female predominance (142 patients, 53.6%). An intertrochanteric fracture was the most common diagnosis (168, 63.4%), followed by neck-of-femur (81, 30.6%) and subtrochanteric (16, 6.0%) fractures. Closed reduction and internal fixation was the most frequently performed procedure (150, 56.6%), followed by bipolar hemiarthroplasty (64, 24.2%), open reduction and internal fixation (44, 16.6%), and total hip replacement (7, 2.6%). Most patients were ASA physical status II (142, 53.6%) or III (70, 26.4%). All patients sustained an isolated hip injury (AIS 3); the ISS was 3 in 262 patients (98.9%), with three patients (1.1%) having an ISS ≥12. Hypertension (64, 24.2%) and diabetes mellitus (57, 21.5%) were the most prevalent comorbidities. When examined by anesthetic technique, the groups were broadly comparable, with no difference in ASA physical status (p = 0.470) or comorbidity count (p = 0.080); however, patients who received CSE anesthesia were significantly younger (median 73 years vs. 78 years for SAB; p = 0.001) and had a higher prevalence of coronary artery disease (17.1% vs 5.1%; p = 0.012), with a non-significant tendency towards more femoral-neck fractures (Table 1).

**Table 1:** Baseline demographic and clinical characteristics of the study cohort, overall and by anesthetic technique (N = 265) Data are n (%) unless otherwise stated. Continuous variables (age, number of comorbidities) were compared with the Kruskal-Wallis test (H statistic, two degrees of freedom) and categorical variables with the Pearson chi-square test (χ² statistic). Owing to the small size of the general-anesthesia group, several comparisons had low expected cell counts, and the corresponding p-values should be interpreted with caution. The Abbreviated Injury Scale was 3 in all patients. SAB: subarachnoid block; CSE: combined spinal-epidural anesthesia; GA: general anesthesia; ASA: American Society of Anesthesiologists physical status; CRIF: closed reduction and internal fixation; ORIF: open reduction and internal fixation; THR: total hip replacement; COPD: chronic obstructive pulmonary disease; ISS: Injury Severity Score; IQR: interquartile range

Characteristic	Total (N = 265)	SAB (N = 156)	CSE (N = 76)	GA (N = 33)	Test statistic; p-value
Age, years, mean ± SD	76.7 ± 7.9	78.1 ± 7.9	74.8 ± 7.3	74.2 ± 7.9	H(2) = 13.20; p = 0.001
Female sex, n (%)	142 (53.6)	88 (56.4)	38 (50.0)	16 (48.5)	χ²(2) = 1.24; p = 0.538
Fracture type, n (%)					
Intertrochanteric	168 (63.4)	108 (69.2)	40 (52.6)	20 (60.6)	χ²(4) = 8.73; p = 0.068
Neck of femur	81 (30.6)	42 (26.9)	30 (39.5)	9 (27.3)	
Subtrochanteric	16 (6.0)	6 (3.8)	6 (7.9)	4 (12.1)	
Surgical procedure, n (%)					
CRIF	150 (56.6)	92 (59.0)	38 (50.0)	20 (60.6)	χ²(6) = 8.36; p = 0.213
Bipolar hemiarthroplasty	64 (24.2)	36 (23.1)	20 (26.3)	8 (24.2)	
ORIF	44 (16.6)	27 (17.3)	13 (17.1)	4 (12.1)	
THR	7 (2.6)	1 (0.6)	5 (6.6)	1 (3.0)	
ASA physical status, n (%)					
I or II	188 (70.9)	107 (68.6)	58 (76.3)	23 (69.7)	χ²(2) = 1.51; p = 0.470
III or IV	77 (29.1)	49 (31.4)	18 (23.7)	10 (30.3)	
ISS ≥ 12, n (%)	3 (1.1)	1 (0.6)	0 (0.0)	2 (6.1)	χ²(2) = 8.37; p = 0.015
No. of comorbidities, median (IQR)	0 (0–1)	0 (0–1)	1 (0–2)	1 (0–1)	H(2) = 5.06; p = 0.080
Comorbidities, n (%)					
Hypertension	64 (24.2)	36 (23.1)	21 (27.6)	7 (21.2)	χ²(2) = 0.76; p = 0.685
Diabetes mellitus	57 (21.5)	31 (19.9)	19 (25.0)	7 (21.2)	χ²(2) = 0.80; p = 0.671
Coronary artery disease	24 (9.1)	8 (5.1)	13 (17.1)	3 (9.1)	χ²(2) = 8.90; p = 0.012
COPD	14 (5.3)	9 (5.8)	2 (2.6)	3 (9.1)	χ²(2) = 2.10; p = 0.350
Prior stroke	11 (4.2)	5 (3.2)	5 (6.6)	1 (3.0)	χ²(2) = 1.58; p = 0.454
Renal disease	11 (4.2)	3 (1.9)	6 (7.9)	2 (6.1)	χ²(2) = 4.93; p = 0.085
Thyroid disorder	7 (2.6)	4 (2.6)	3 (3.9)	0 (0.0)	χ²(2) = 1.40; p = 0.496
Malignancy	5 (1.9)	3 (1.9)	2 (2.6)	0 (0.0)	χ²(2) = 0.86; p = 0.649
Dementia	4 (1.5)	0 (0.0)	3 (3.9)	1 (3.0)	χ²(2) = 5.94; p = 0.051
Heart failure	3 (1.1)	2 (1.3)	1 (1.3)	0 (0.0)	χ²(2) = 0.43; p = 0.806
Atrial fibrillation	3 (1.1)	1 (0.6)	2 (2.6)	0 (0.0)	χ²(2) = 2.24; p = 0.326
Asthma	3 (1.1)	3 (1.9)	0 (0.0)	0 (0.0)	χ²(2) = 2.12; p = 0.346
Seizure disorder	1 (0.4)	0 (0.0)	1 (1.3)	0 (0.0)	χ²(2) = 2.50; p = 0.287

Anesthetic technique and hospital length of stay

SAB was the predominant technique (156, 58.9%), followed by CSE anesthesia (76, 28.7%) and GA (33, 12.5%). The overall median hospital length of stay was five days (IQR 3-7; range 2-41). Length of stay differed significantly across anesthetic techniques (Kruskal-Wallis H = 199.26, df = 2, p < 0.001), being shortest after SAB (median four days, IQR 3-5), intermediate after GA (six days, IQR 6-7), and longest after CSE anesthesia (nine days, IQR 7.5-11). All three pairwise comparisons were significant on Dunn’s post-hoc testing with Bonferroni adjustment: CSE versus SAB (p < 0.001), GA versus SAB (p < 0.001), and CSE versus GA (p = 0.002) (Table 2).

**Table 2:** Hospital length of stay by anesthetic technique Overall difference across techniques: Kruskal-Wallis test, H = 199.26 (df = 2), p < 0.001. Dunn’s post-hoc pairwise comparisons (Bonferroni-adjusted): CSE vs SAB z = 13.71, p < 0.001; GA vs SAB z = 6.53, p < 0.001; CSE vs GA z = 3.20, p = 0.002. LOS: length of stay; IQR: interquartile range

Anesthetic technique	n (%)	Median LOS (IQR), days	Range, days
Subarachnoid block (SAB)	156 (58.9)	4 (3–5)	2–6
Combined spinal–epidural (CSE)	76 (28.7)	9 (7.5–11)	5–41
General anesthesia (GA)	33 (12.5)	6 (6–7)	6–7
Overall	265 (100)	5 (3–7)	2–41

Anesthetic technique, ICU stay, and postoperative adverse events

Sixteen patients (6.0%) required ICU admission, more frequently after CSE anesthesia (10/76, 13.2%) than after GA (2/33, 6.1%) or SAB (4/156, 2.6%; Fisher’s exact p = 0.006). ICU length of stay could be determined in 15 of these patients and was longest in the CSE group (median eight days, IQR 5.25-19.5; n = 10) compared with SAB (two days; n = 3) and GA (1.5 days; n = 2), although this comparison rests on very small numbers (Kruskal-Wallis p = 0.040).

A postoperative adverse event of any type occurred in 22 patients (8.3%), and its frequency differed across techniques, being highest after CSE (13/76, 17.1%), intermediate after GA (3/33, 9.1%), and lowest after SAB (6/156, 3.8%; p = 0.002). Among individual complications, respiratory events (p = 0.018) and acute kidney injury (p = 0.004) were significantly more frequent in the CSE group, in which the majority of all complications occurred; the remaining individual events were infrequent and did not differ significantly between techniques. In-hospital mortality (four patients, 1.5%) and 30-day readmission (five patients, 1.9%) did not differ by technique (p = 0.223 and p = 0.511, respectively) (Table 3).

**Table 3:** Postoperative outcomes and adverse events, overall and by anesthetic technique Data are n (%). p-values from Fisher’s exact test (Freeman-Halton extension for the 3×2 tables); no test statistic is defined for Fisher’s exact test. Comparisons of the individual, low-frequency complications are exploratory and were not adjusted for multiple testing. ICU length of stay (available for 15 of 16 ICU patients) was compared separately using the Kruskal-Wallis test (H = 6.42, df = 2, p = 0.040). SAB: subarachnoid block; CSE: combined spinal-epidural anesthesia; GA: general anesthesia

Outcome / adverse event	SAB (N = 156)	CSE (N = 76)	GA (N = 33)	Total (N = 265)	p-value
ICU admission	4 (2.6)	10 (13.2)	2 (6.1)	16 (6.0)	0.006
Any postoperative adverse event	6 (3.8)	13 (17.1)	3 (9.1)	22 (8.3)	0.002
In-hospital death	1 (0.6)	2 (2.6)	1 (3.0)	4 (1.5)	0.223
Respiratory events	1 (0.6)	5 (6.6)	0 (0.0)	6 (2.3)	0.018
Acute kidney injury	0 (0.0)	5 (6.6)	0 (0.0)	5 (1.9)	0.004
Myocardial infarction	1 (0.6)	1 (1.3)	0 (0.0)	2 (0.8)	0.654
Surgical site infection	1 (0.6)	2 (2.6)	0 (0.0)	3 (1.1)	0.498
Urinary retention	1 (0.6)	1 (1.3)	1 (3.0)	3 (1.1)	0.223
Postoperative atrial fibrillation	1 (0.6)	1 (1.3)	0 (0.0)	2 (0.8)	0.654
Postoperative stroke	0 (0.0)	2 (2.6)	0 (0.0)	2 (0.8)	0.168
Delirium	0 (0.0)	0 (0.0)	1 (3.0)	1 (0.4)	0.125
Sepsis	0 (0.0)	1 (1.3)	0 (0.0)	1 (0.4)	0.411
Deep vein thrombosis	0 (0.0)	0 (0.0)	1 (3.0)	1 (0.4)	0.125
Postoperative seizure	1 (0.6)	0 (0.0)	0 (0.0)	1 (0.4)	1.000
30-day readmission	2 (1.3)	2 (2.6)	1 (3.0)	5 (1.9)	0.511

Patient factors associated with hospital length of stay

On univariable (Mann-Whitney) testing, a significantly longer hospital stay was associated with coronary artery disease (p < 0.001), renal disease (p = 0.017), and prior stroke (p = 0.036); the association with diabetes mellitus was of borderline significance (p = 0.053). Hypertension, atrial fibrillation, heart failure, chronic obstructive pulmonary disease (COPD), asthma, thyroid disorder, seizure disorder, and malignancy were not significantly associated with length of stay, and length of stay did not differ by gender (p = 0.226). Length of stay also did not differ across ASA physical-status categories (Kruskal-Wallis p = 0.419) (Table 4).

**Table 4:** Univariable association of patient factors with hospital length of stay z values are from the two-sample Wilcoxon rank-sum (Mann-Whitney U) test comparing hospital length of stay between patients with and without each binary factor; a negative z denotes a longer stay in the presence of the factor. ASA physical status was compared across categories I-IV using the Kruskal-Wallis test (H statistic, three degrees of freedom). Liver disease was not analyzed (no affected patients). COPD: chronic obstructive pulmonary disease; ASA: American Society of Anesthesiologists

Factor	n with factor (%)	Test statistic	p-value
Coronary artery disease	24 (9.1)	z = −3.46	<0.001
Renal disease	11 (4.2)	z = −2.38	0.017
Prior stroke	11 (4.2)	z = −2.10	0.036
Diabetes mellitus	57 (21.5)	z = −1.94	0.053
Hypertension	64 (24.2)	z = −1.46	0.145
Thyroid disorder	7 (2.6)	z = −1.61	0.108
Asthma	3 (1.1)	z = 1.55	0.120
Seizure disorder	1 (0.4)	z = −1.52	0.128
Atrial fibrillation	3 (1.1)	z = −1.01	0.314
Heart failure	3 (1.1)	z = 0.51	0.609
Malignancy	5 (1.9)	z = −0.29	0.774
COPD	14 (5.3)	z = −0.01	0.993
Female gender	142 (53.6)	z = −1.21	0.226
ASA physical status (I–IV)	—	H = 2.83	0.419

Adjusted analysis

In a multivariable negative-binomial model, the association between anesthetic technique and hospital length of stay persisted after adjustment for age, sex, ASA physical status, fracture type, and the number of comorbidities: relative to SAB, length of stay was longer with CSE anesthesia (adjusted IRR 2.84, 95% CI 2.51-3.22) and with GA (IRR 1.72, 95% CI 1.44-2.05; both p < 0.001). Among the other covariates, only a neck-of-femur fracture was independently associated with a longer stay (IRR 1.19, 95% CI 1.05-1.35, p = 0.006), with a borderline contribution from the number of comorbidities (p = 0.056); age, sex, and ASA grade were not significant (Table 5). A median (quantile) regression model fitted with the same covariates as a sensitivity analysis gave concordant results, with an adjusted increase in median length of stay of 5.3 days for CSE and 2.5 days for GA relative to SAB.

**Table 5:** Multivariable negative-binomial model of hospital length of stay (N = 265) Estimates are from a multivariable negative-binomial regression model. Wald z is the test statistic for each coefficient. Reference categories: subarachnoid block, female sex, intertrochanteric fracture. The dispersion parameter was estimated by maximum likelihood (the negative-binomial model was preferred to the Poisson owing to overdispersion). IRR: incidence-rate ratio; CI: confidence interval; IT: intertrochanteric; NOF: neck of femur; SAB: subarachnoid block; CSE: combined spinal-epidural anesthesia; GA: general anesthesia; ASA: American Society of Anesthesiologists physical status

Covariate	Adjusted IRR	95% CI	Wald z	p-value
Technique: CSE (vs. SAB)	2.84	2.51–3.22	16.36	<0.001
Technique: GA (vs. SAB)	1.72	1.44–2.05	5.94	<0.001
Fracture: neck-of-femur (vs. intertrochanteric)	1.19	1.05–1.35	2.77	0.006
Fracture: subtrochanteric (vs. intertrochanteric)	1.08	0.86–1.36	0.65	0.513
Number of comorbidities (per one)	1.05	1.00–1.11	1.91	0.056
ASA grade (per level)	1.04	0.96–1.13	1.05	0.292
Age (per year)	1.00	1.00–1.01	1.14	0.256
Male sex (vs. female)	0.99	0.89–1.11	-0.17	0.865

## Discussion

In this retrospective cohort of 265 geriatric patients undergoing acute hip fracture surgery, the anesthetic technique was strongly associated with hospital length of stay: the median stay was shortest after SAB, intermediate after GA, and longest after CSE anesthesia, and this gradient persisted after adjustment for age, sex, ASA status, fracture type, and comorbidity burden. ICU admission and postoperative adverse events followed the same pattern, being concentrated in the CSE group, whereas in-hospital mortality (1.5%) and 30-day readmission (1.9%) were low and did not differ by technique. Among patient factors, coronary artery disease, renal disease, and prior stroke were each associated with a longer stay.

These associations are best interpreted in the light of how the anesthetic technique was selected. In routine practice, the approach is chosen by the attending anesthesiologist according to the patient’s condition and the planned operation rather than at random. Importantly, the three groups were broadly comparable at baseline, and patients who received CSE anesthesia were not older or of higher ASA physical status than the other groups, but they had a higher prevalence of coronary artery disease and a non-significant tendency towards more femoral-neck fractures. Crucially, the length-of-stay gradient was only marginally attenuated by adjustment for these measured characteristics, indicating that recorded differences in age, comorbidity, ASA grade, and fracture type explain very little of it. The persistent gradient is therefore more plausibly attributable to the indications for and the care pathways associated with each technique, for example, the use of an indwelling epidural catheter for prolonged analgesia and the longer, monitored stays that accompany it, together with unmeasured determinants of technique selection and institutional discharge practices, rather than to a direct effect of the anesthetic agent on recovery. The narrow, almost uniform stay durations within the SAB and GA groups further point to a strong institutional and pathway component.

This interpretation is supported by the best available randomized evidence. In the large multicenter Regional versus General Anesthesia for Promoting Independence after Hip Fracture (REGAIN) trial and its prespecified one-year analysis, SAB and GA produced similar long-term outcomes after hip fracture surgery: survival at up to one year did not differ between arms, and the composite outcomes of death or new inability to walk and death or new nursing-home admission at one year were also comparable [10,11]. A systematic review and meta-analysis confined to randomized controlled trials reached the same conclusion, finding no significant difference between GA and RA in perioperative mortality, intraoperative complications, or hospital length of stay [12]. Because randomization removes confounding by indication, the absence of any outcome difference in these trials argues strongly that the marked between-technique differences observed in observational cohorts such as ours are driven by which patients receive which technique rather than by the technique itself. Our own finding of similar in-hospital mortality across techniques is concordant with this randomized evidence and with the broader meta-analytic literature, which has not demonstrated a consistent mortality benefit for either approach [4-6]; where pooled observational data have suggested a lower in-hospital mortality with RA, the same analyses have found no corresponding difference in 30-day mortality, postoperative pneumonia, or delirium and have attributed the in-hospital signal largely to a single very large study [13].

The contrast between randomized and observational findings is instructive when interpreting our own data. Large population-based and registry studies have repeatedly reported associations between RA and better short-term outcomes that are not reproduced in randomized trials. A nationwide Taiwanese cohort of 17,189 patients found no association between anesthetic type and 30-day mortality but a lower rate of 30-day all-cause and surgical-site-infection readmission with RA [14], while two recent analyses of the American College of Surgeons National Surgical Quality Improvement Program (NSQIP) reported lower rates of a composite of death, myocardial infarction, or stroke with spinal or peripheral-nerve-block techniques than with GA, with the benefit concentrated almost entirely in the highest-risk patients (ASA physical status IV-V) [15,16]. Such subgroup-dependent associations are precisely the pattern expected when sicker patients are selectively directed towards or away from a particular technique, and they reinforce the view that the technique outcome associations in non-randomized data, including the length-of-stay gradient in our cohort, reflect patient selection and care pathways rather than a pharmacological effect of the anesthetic.

The clustering of acute kidney injury and respiratory events in the CSE group in our cohort similarly cannot be attributed to the technique. Although a recent meta-analysis suggested a lower incidence of acute kidney injury with SAB compared with GA [17], the events in our study were few and occurred in the group with the highest prevalence of pre-existing coronary and renal disease; with only five cases of acute kidney injury, no causal inference is possible. The physiological differences between techniques that might be expected to drive such complications do not translate reliably into divergent outcomes: in a randomized trial comparing desflurane-based balanced anesthesia, propofol-based total intravenous anesthesia, and SAB, the spinal group required significantly fewer intraoperative vasopressors and less fluid, yet postoperative pulmonary, cardiac, and neurological complications, inflammatory markers, and mortality were comparable across all three groups [18]. Likewise, although RA techniques have been proposed to reduce postoperative delirium, a recent randomized trial found no reduction in delirium with RA compared with GA [19], underscoring that differences in complication rates between techniques observed in non-randomized data should be interpreted with caution.

Length of stay, our primary outcome, warrants particular scrutiny because it is especially susceptible to non-pharmacological influences. A retrospective cohort study from Portugal found no difference in either 30-day mortality or length of stay between GA and RA and explicitly attributed residual variation in length of stay to non-clinical factors like surgeon-dependent discharge criteria, the availability of downstream rehabilitation and continued-care beds, and patients’ social circumstances rather than to the anesthetic [20]. These observations mirror our own: the narrow, almost uniform stay durations within the SAB and GA groups, set against the long and variable stays after CSE anesthesia, are far more consistent with technique-specific care pathways, notably the use of an indwelling epidural catheter for prolonged analgesia and the monitored admissions that accompany it, rather than with any direct effect of the anesthetic on recovery. Conversely, where RA forms part of a deliberate pathway intended to accelerate recovery, length of stay may shorten: a recent three-way comparison reported earlier mobilization and shorter hospital stays in patients who received an erector spinae plane block rather than GA or SAB for proximal femoral nailing [21]. Taken together, these data indicate that the relationship between anesthetic technique and length of stay may be influenced by the perioperative pathway in which the technique is embedded rather than by the anesthetic itself.

Within these constraints, our study contributes contemporary real-world data from a high-volume trauma center and shows that, in this geriatric population, anesthetic technique is closely tied to patient and operative characteristics. Length of stay was also independently prolonged by neck-of-femur fracture and, to a borderline degree, by greater comorbidity, identifying patient groups in whom longer admissions can be anticipated and resources planned. The practical implication aligns with current consensus and randomized evidence: because the choice between neuraxial and GA was not associated with a difference in survival in our study and has not consistently demonstrated mortality benefit in randomized studies, the technique can reasonably be individualized to the patient’s physiology, anticoagulation status, and the operative plan rather than selected in the expectation of improving mortality [10,11].

Strengths and limitations

Strengths of this study include complete capture of in-hospital outcomes, a focus on the geriatric trauma population, and the use of distribution-appropriate non-parametric analyses with a multivariable model and a concordant quantile-regression sensitivity analysis. Several limitations must be acknowledged. First and most importantly, the retrospective, non-randomized design makes confounding by indication unavoidable, and the measured covariates cannot fully capture differences in baseline severity, frailty, or anticoagulation that influence technique selection; the length-of-stay associations are therefore hypothesis-generating and not causal. Second, the study is subject to selection bias inherent in a single-center retrospective design: the cohort comprised only those geriatric patients who reached operative fixation at a tertiary-level trauma center and thus does not represent patients managed non-operatively, those who died before surgery, or those treated elsewhere, and any patients with incomplete or untraceable records would not have entered the analysis. Third, because the data were generated for clinical rather than research purposes, the study is exposed to information bias. This is especially relevant to the higher adverse-event and ICU rates observed in the CSE group, because patients with longer, monitored admissions have a greater opportunity for events to be detected and recorded (surveillance bias), which may have exaggerated the apparent association between technique and complications. Fourth, length of stay is also shaped by non-clinical factors such as discharge pathways, social circumstances, and operating-list scheduling that were not recorded. Fifth, ICU length of stay and individual complications were too infrequent for reliable comparison, and the exploratory event-level p-values were not corrected for multiple testing. Sixth, mortality was ascertained in-hospital only, and true 30-day and longer-term outcomes were not available. Finally, comorbidity was captured as individual conditions and a simple count rather than a validated index such as the Charlson Comorbidity Index.

## Conclusions

In this retrospective cohort of geriatric hip fracture patients, anesthetic technique was associated with hospital length of stay, ICU admission, and postoperative adverse events, with the highest rates observed in the CSE group and the lowest in the SAB group. In-hospital mortality was low and did not differ between techniques. As this was an observational study, these findings should be interpreted as associations rather than causal effects. Differences in patient selection, perioperative care pathways, and discharge practices may have contributed to the observed outcomes. The comparable baseline characteristics across groups and the lack of mortality differences are consistent with evidence from randomized trials showing no clear superiority of one anesthetic technique over another. Therefore, anesthetic choice for geriatric hip fracture surgery should be individualized based on patient characteristics and surgical requirements. Prospective studies incorporating perioperative care and discharge-related variables are needed to better understand the mechanisms underlying these associations.
